# Chicken Gut Microbiota Responses to Dietary *Bacillus subtilis* Probiotic in the Presence and Absence of *Eimeria* Infection

**DOI:** 10.3390/microorganisms10081548

**Published:** 2022-07-31

**Authors:** Fareed Uddin Memon, Yunqiao Yang, Geyin Zhang, Imdad Hussain Leghari, Feifei Lv, Yuhan Wang, Farooque Laghari, Farooque Ahmed Khushk, Hongbin Si

**Affiliations:** 1 State Key Laboratory for Conservation and Utilization of Subtropical Agro-Bioresources, College of Animal Science and Technology, Guangxi University, Nanning 530004, China; fareedjanm@gmail.com (F.U.M.).; yyqop01@163.com (Y.Y.); 18838516358@163.com (G.Z.); 1918393033@st.gxu.edu.cn (F.L.); 1918393052@st.gxu.edu.cn (Y.W.); 2 Department of Poultry Husbandry, Faculty of Animal Husbandry and Veterinary Sciences, Sindh Agriculture University, Tando Jam 70060, Pakistan; ihleghari@sau.edu.pk (I.H.L.); brainboss48@gmail.com (F.A.K.); 3 Department of Animal Production and Environment Control, College of Animal Sciences and Technology, Southeast Agriculture University, Harbin 150030, China; laghari_farooque@yahoo.com

**Keywords:** *Bacillus subtilis* probiotic, chicken, gut, *Eimeria*, microbiome analysis

## Abstract

Coccidiosis is a well-known poultry disease that causes the severe destruction of the intestinal tract, resulting in reduced growth performance and immunity, disrupted gut homeostasis and perturbed gut microbiota. Supplementation of probiotics were explored to play a key role in improving growth performance, enhancing innate and adaptive immunity, maintaining gut homeostasis and modulating gut microbiota during enteric infection. This study was therefore designed to investigate the chicken gut whole microbiota responses to *Bacillus subtilis* (*B. subtilis*) probiotic feeding in the presence as well as absence of *Eimeria* infection. For that purpose, 84 newly hatched chicks were assigned into four groups, including (1) non-treated non-challenged control group (CG − ET), (2) non-treated challenged control group (CG + ET), (3) *B. subtilis*-fed non-challenged group (BS − ET) and (4) *B. subtilis*-fed challenged group (BS + ET). CG + ET and BS + ET groups were challenged with *Eimeria tenella* (*E. tenella*) on 21 day of housing. Our results for Alpha diversity revealed that chickens in both infected groups (CG + ET and BS + ET) had lowest indexes of Ace, Chao 1 and Shannon, while highest indexes of Simpson were found in comparison to non-challenged groups (CG − ET and BS − ET). *Firmicutes* was the most affected phylum in all experimental groups following *Proteobacteria* and *Bacteroidota*, which showed increased abundance in both non-challenged groups, whereas *Proteobacteria* and *Bacteroidota* affected both challenged groups. The linear discriminant analysis effect size method (lEfSe) analysis revealed that compared to the CG + ET group, supplementation of probiotic in the presence of *Eimeria* infection increased the abundance of some commensal genera, included *Clostridium sensu stricto 1*, *Corynebacterium*, *Enterococcus*, *Romboutsia*, *Subdoligranulum*, *Bacillus*, *Turicibacter* and *Weissella*, with roles in butyrate production, anti-inflammation, metabolic reactions and the modulation of protective pathways against pathogens. Collectively, these findings evidenced that supplementation of *B. subtilis* probiotic was positively influenced with commensal genera, thereby alleviating the *Eimeria*-induced intestinal disruption.

## 1. Introduction

Avian coccidiosis is one of the major problems in the chicken industry, which is associated with approximately USD 14.5 billion of economic loss globally, including production, prevention and treatment losses [1]. Avian coccidiosis is a protozoan parasitic disease caused by several species of *Protista* belonging to the phylum *Apicomplexa* that mainly affects the intestinal tract of birds [2]. The intestinal proliferation of *Eimeria* leads to the severe destruction of epithelial cells and necrosis, which results in bloody diarrhea, weight loss and eventually death [3]. Seven different species of *Eimeria* (*E. maxima*, *E. mitis*, *E. necatrix*, *E. acervulina*, *E. paraecox*, *E. brunetti* and *E. tenella*) were recognized for coccidiosis in chickens that occupy and invade different parts of intestine. *E. tenella* was found as one of the most devastating species, which resides in the cecum of chicken and causes destruction of mucosa through villi epithelial cells, resulting in severe epithelial damage, bloody feces, reduced weight gain, decreased feed efficiency and ultimate death [4]. Various control measures, including chemical and synthetic drugs, have been implemented to overcome *Eimeria* infection in chickens. However, with the passage of time, the *Eimeria* species developed resistance due to repeated and prolonged use of these drugs [5]. Another strategy for controlling coccidiosis is the use of attenuated and non-attenuated vaccines, but the poor use of these vaccines may cause disease reversion [6]. Therefore, the current circumstances demand alternative and safe control strategies to overcome the disease [7].

Supplementation of probiotics (live non-pathogenic microbes) maintains the gut microbiota and influences the overall performance of chickens [8]. World Health Organization (WHO) and Food and Agriculture Organization of the United Nations (FAO) defined probiotics as “live non-pathogenic micro-organisms, which when administered in adequate amount confer a health benefits on host” [9]. A variety of microorganisms may be used as probiotics. The most common genera belong to the bacteria but some yeasts and molds may also be used as probiotics [10]. Microbial genera that are commonly used as probiotics include species of *Bacillus*, *Enterococcus*, *Lactobacillus*, *Lactococcus*, *Streptococcus*, *Bifidobacterium*, *Pedicoccus*, *Leuconostoc* and *Propionibacterium* in bacterial genera, *Saccharomyces* in yeasts and *Aspergillus* in molds [11].

*Bacillus-*based probiotics are widely used as alternatives to antibiotics in animal and chicken feed due to their capacity to form spores, which produce resistance against high temperatures, pH, bile and enzymes encountered in the gastrointestinal tract (GIT), withstand harsh conditions and confer health benefits to the host [12,13]. Previous studies showed that supplementation with *Bacillus*-based probiotics had positive effects on the growth performance, immunity, gut homeostasis and microbiota of fishes, piglets and chickens [14,15,16]. Hung et al. [17] reported that feeding of *Bacillus*-based probiotic to chickens improved performance and beneficially modulated the composition of microflora, which markedly increased the abundance of *Lactobacilli* and decreased the *Coliform*. Jacquier et al. [18] observed that the supplementation of *Bacillus subtilis* 29,784 increased some abundance bacterial genera with roles in the production of butyrate and linoleic acid.

Next-generation sequencing technology opened the doors for scientists to explore the gut microbiome on a deeper and broader level [19]. Researchers expressed interest in using high-throughput sequencing technology to identify and classify the chicken gut microbes because conventional molecular ecology techniques such as denaturing gradient gel electrophoresis (DGGE) fingerprints can only detect a smaller microbial population and it is difficult to identify the structure and composition of microflora using such techniques [20,21]. Therefore, in this study, a MiSeq high-throughput sequencing analysis was performed to reveal the underlying mechanisms of *B. subtilis* probiotic on chicken gut microbiota responses in the absence and presence of *Eimeria* infection.

## 2. Materials and Methods

### 2.1. Probiotic and Isolation of E. tenella

Commercial *Bacillus*-based probiotic was used in this study, which was purchased from Kangjialong Feed Co., Ltd. Nanning, China (201906157), and contained 1 × 10^8^ cfu/g of *B. subtilis*, which was supplemented in feed at the rate of 1 g/kg of feed.

The utilized *E. tenella* strain in this study was collected from the field (Guangxi, China) from infected chicken’s cecum. Sporulation and purification of collected oocysts were carried out according to the protocols described in our previous study [22].

### 2.2. Experimental Design

Eighty four newly hatched Chinese native yellow breed chicks were purchased from a commercial hatchery and housed in twelve battery cages with ad libitum feed and water throughout the 28 days trial. All animal experimental protocols were approved by the Animal Care and Use Committee of Guangxi University, Nanning, China (approval code is Gxu-2019-180) and the studies were carried out in accordance with their guidelines.

Upon arrival, chicks were randomly assigned into the following four groups (21 chicks/group): (1) control group non-treated non-challenged (CG − ET), chickens fed basal diet but not challenged with *E. tenella*, (2) control group non-treated challenged (CG + ET), chickens fed basal diet and challenged with *E. tenella* on day 21 of age, (3) *B. subtilis*-fed non-challenged group (BS − ET), chickens fed diets supplemented with *B. subtilis* probiotic, but not challenged with *E. tenella*, and (4) *B. subtilis*-fed challenged group (BS + ET), chickens fed diets supplemented with *B. subtilis* probiotic and challenged with *E. tenella* on day 21 of age. The innoculation dose of *E. tenella* sporulated oocysts was 6 × 10^4^. In order to provide the same management stress, chickens in groups CG − ET and BS − ET were gavaged normal saline. To avoid the transmission of infection, non-challenged and challenged chickens were housed in different temperature-controlled rooms (32 to 34 °C for the 1st week and then decreased by 2 °C/week) with 60 to 80% humidity and 23 h light. All groups were examined for performance and clinical indexes, such as pre-infection and post-infection body weights, bloody diarrhea scores, oocyst counting in feces and cecal lesion scores and the obtained results were merged with our previous study [23].

### 2.3. Sample Collection, Extraction of DNA and PCR Amplification

Three fresh fecal samples (one from each replicate cage) were collected on day 28 of age and collected samples were used to extract the intestinal bacterial genomic DNA using E.Z.N.A^®^ Soil DNA Kit (Omega Bio-Tek, Norcross, GA, USA) following the recommendations of manufacturers. Concentration and purity of the DNA were examined using NanoDrop 2000 spectrophotometer (260–280 nm ratios) and 1% agarose gel electrophoresis. The V3–V4 hypervariable region of 16S rRNA gene was amplified by PCR using fusion primers [Forward primer: 520 (5-ACTCCTACGGGAGGCAGCAG-3) and reverse primer: 806 (5-GGACTACHVGGGTWTCTAAT-3)]. The thermal cycling program consisted of initial denaturation for 3 m at 95 °C, followed by 27 cycles of denaturation for 30 s at 95 °C, annealing for 30 s at 55 °C and extension for 45 s at 72 °C, with final extension for 7 m at 72 °C. Each sample was repeated 3 times. The components of PCR reaction contained 4 µL of 5× Fast Pfu buffer, 2 µL of 2.5 Mm dNTPs, 0.8 µL of each primer, 0.4 µL of Fast Pfu Polymerase, 0.2 µL of BSA and 10 ng of template DNA. An electrophoresis chamber was used to run the PCR products on 2% and the purification of the PCR product was carried out using AxyPrep DNA Gel Extraction Kit (Axygen Bioscience, Union City, CA, USA) following the manufacturer’s manual. Following purification, the amplicons were used for library preparation and pyrosequencing. The NEB-Next^®^ Ultra™ DNA Library Preparation Kit (New England Bio-labs, Ipswich, MA, USA) was used to generate the sequencing libraries and the sequencing of libraries was performed using the Illumina MiSeq PE 300 platform (Illumina, Inc., San Diego, CA, USA).

### 2.4. MiSeq Sequencing Analyses

In order to process the sequencing data, the QIIME (Quantitative Insights Into Microbial Ecology) pipeline was employed [24]. In this step, the sequences matched with barcodes were allocated to respective samples and the valid sequences were considered. Filtration of low quality sequences was carried out by removing the sequences that had a length of below 50 bp, Phred scores below 20 and contained ambiguous bases. Paired-end reads were generated using FLASH [25]. Following chimeric sequences detection by UCHIME, the remaining high quality sequences were clustered in OTUs (operational taxonomic units) with 97% sequence similarity cutoff by using UPARSE software [26] and the representive sequences were selected from each OTU. Taxonomical classification of OTUs was conducted using the RDP Classifier algorithm against SILVA database. The alpha diversities were measured based on the values of Ace, Chao 1, Shannon and Simpson. Structural variations of microbial communities and abundances of taxa at the levels of phylum and genus were statistically compared among groups by linear discriminant analysis (LDA) and effect size method (lEfSe) in order to assess the differentially abundant taxa across groups [27]. For the LEfSe analysis, the threshold ratio was 0.05. Phylogenetic investigation of communities by the reconstruction of unobserved states (PICRUSt) analysis was performed to evaluate the predicted functional genes based on the abundance at the OTU level [28].

### 2.5. Statistical Analysis

Comparative analysis (of current data) among all groups was assessed by Student’s *t*-test using SPSS version 20.0 (SPSS Inc., Chicago, IL, USA). All results are presented as mean and standard deviation. Significant differences were considered at a *p*-value of 0.05.

## 3. Results

### 3.1. Quality Control Determination of MiSeq Sequencing

A total of 159,319,200 bases were obtained from 12 fecal samples of four groups. After checking the quality and removing the chimeric sequences, 531,054 reads were obtained in total and the average length of the sequences was 422 base pairs. Good’s indexes of all samples were calculated to ensure the adequate sequencing depth and found >0.99 of Good’s coverage in each sample (Table 1), indicating that an estimated 99% of the bacteria in collected fecal samples were captured with MiSeq sequencing.

### 3.2. Alpha Diversity

**Figure 1:**
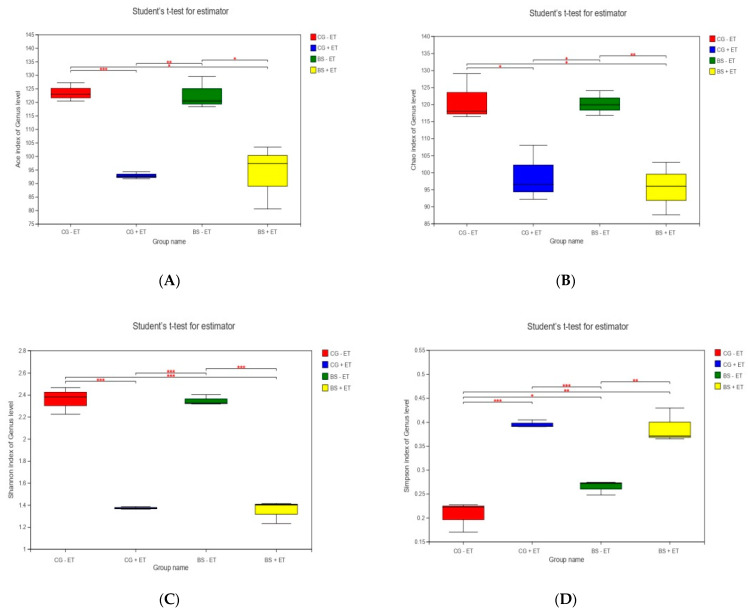
Effects of treatments on alpha diversity index. (**A**) Ace index, (**B**) Chao 1 index, (**C**) Shannon index and (**D**) Simpson index. *, **, *** represent significant difference.

In order to evaluate the alpha diversity, Ace, Chao 1, Shannon and Simpson, which provide the information regarding microbial richness, biodiversity and abundance of species, were calculated. The lowest indexes of Ace, Chao 1 and Shannon, while the highest indexes of Simpson were found in the challenged groups (CG + ET and BS + ET) in comparison to non-challenged groups (CG − ET and BS − ET). However, the indexes of Ace, Chao 1 and Shannon in the comparison of CG − ET versus BS − ET, while Simpson indexes in the comparison of CG + ET versus BS + ET were not affected (Figure 1, Table S1).

**Figure 2:**
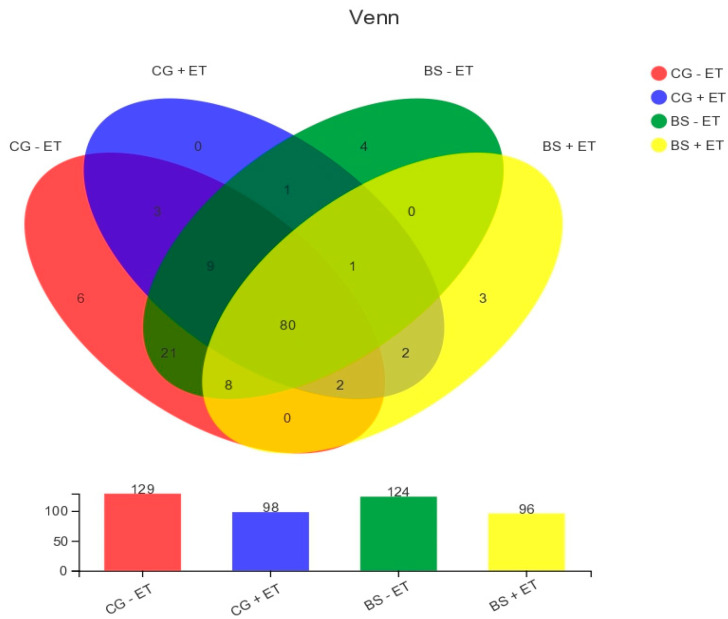
Present Venn diagram shows the all, unique and overlapped OTUs observed in/between treatments.

There were 129, 98, 124 and 96 OTUs observed in groups CG − ET, CG + ET, BS − ET, and BS + ET, respectively, of which 80 were common in all experimental groups. Moreover, a total of 13 unique OTUs were detected within CG − ET, CG + ET, BS − ET, and BS + ET (6, 0, 4, and 3, respectively, Figure 2). 

### 3.3. Effects of Treatments on Bacterial Abundances at Phylum Level

**Figure 3:**
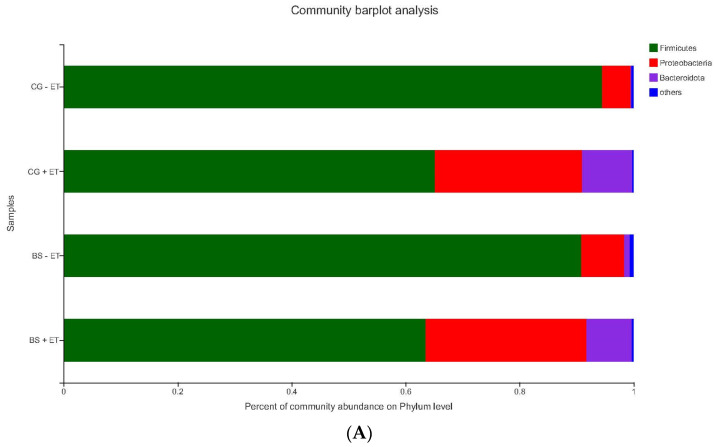

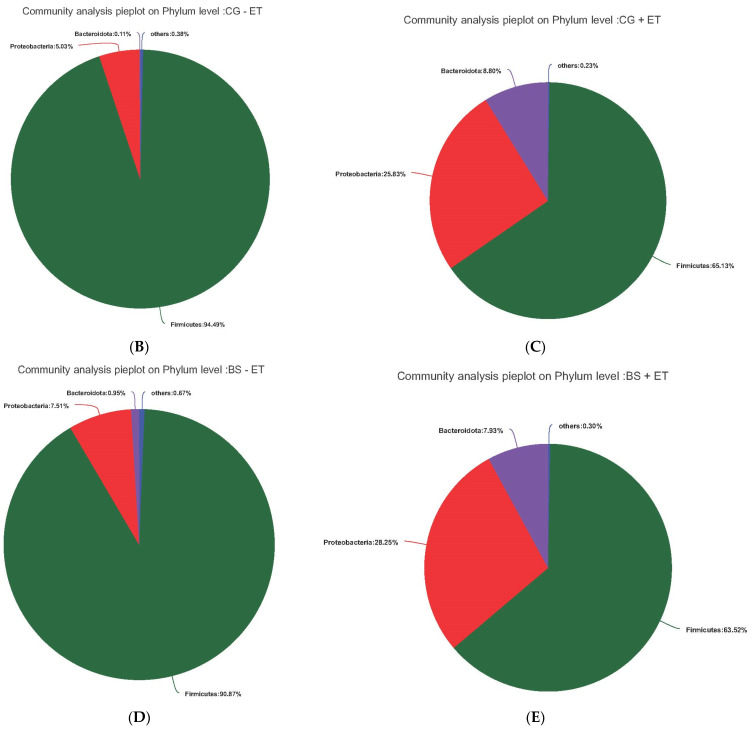
Relative abundances of the taxa at phylum level. (**A**) Relative abundances of the affected taxa at phylum level among all groups. (**B**) Total affected percentage of each phylum in group CG − ET. (**C**) Total affected percentage of each phylum in group CG + ET. (**D**) Total affected percentage of each phylum in group BS − ET. (**E**) Total affected percentage of each phylum in group BS + ET.

*Firmicutes* was the most affected phylum in all experimental groups following *Proteobacteria* and *Bacteroidota*, exhibiting similar abundance between CG − ET and BS − ET groups (94.49% and 90.87%), while it showed relatively lower abundance in CG + ET and BS + ET groups (65.13% and 63.52%)**.** Within these letter groups, CG + ET and BS + ET, the abundances of *Proteobacteria* (25.83% and 28.25%) and *Bacteroidota* (8.80% and 7.93%) were more prominent in relation to the CG − ET (5.03% and 0.11%) and BS − ET (7.51% and 0.95%) groups (Figure 3).

### 3.4. Effects of Treatments on Bacterial Abundances at Genus Level

**Figure 4:**
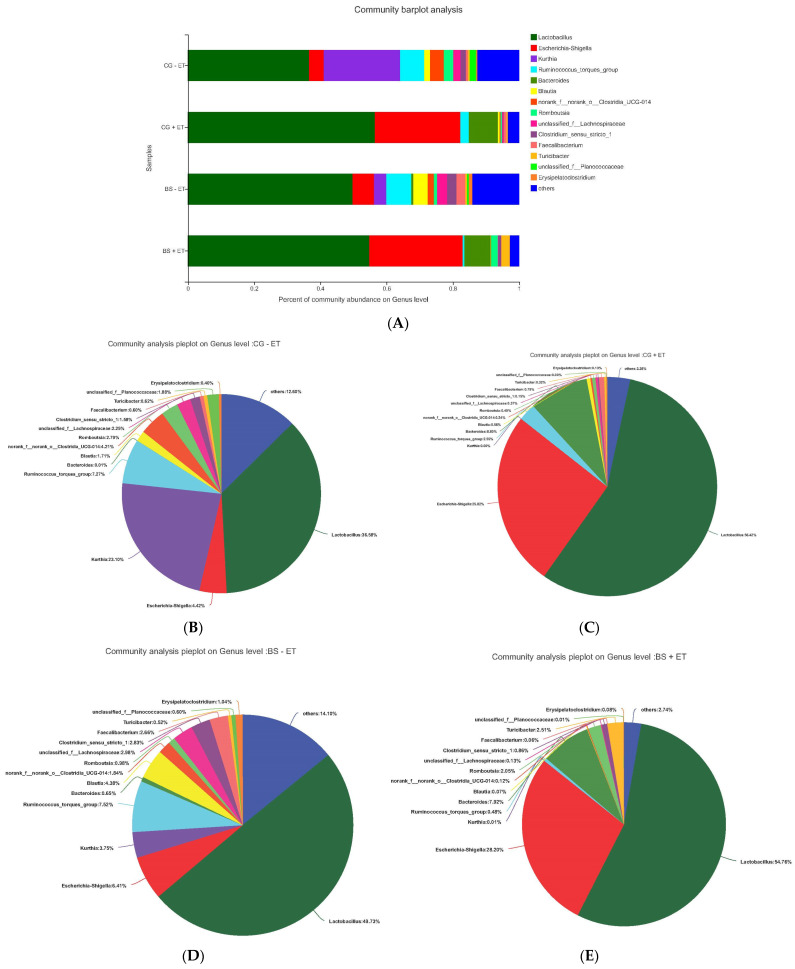
Relative abundances of the taxa at genus level. (**A**) Relative abundances of the affected taxa at genus level among all groups. (**B**) Total affected percentage of each genus in group CG − ET. (**C**) Total affected percentage of each genus in group CG + ET. (**D**) Total affected percentage of each genus in group BS − ET. (**E**) Total affected percentage of each genus in group BS + ET.

We further compared the bacterial composition in feces of all experimental treatments at the genus level (Figure 4), where *Lactobacillus* (belonging to the *Firmicutes* phylum), *Escherichia-Shigella* (belonging to the *Proteobacteria* phylum) and *Bacteroides* were the most affected genera. The relative abundances of *Lactobacillus* accounted for 36.56%, 56.42%, 49.73%, and 54.76; *Escherichia-Shigella* accounted for 4.42%, 25.82%, 6.41%, and 28.20%; and *Bacteroides* accounted for 0.01%, 8.80%, 0.65%, and 7.92% of the total population in groups CG- ET, CG + ET, BS − ET, and BS + ET, respectively, showing clear increased abundance in the presence of *Eimeria* infection and probiotic feeding. In contrast, the declined abundances of *Kurthia*, *Ruminococcus-torques-group*, *norank*-*Clostridia UCG-014* were found in *Eimeria*-infected (CG + ET and BS + ET) groups compared to the CG − ET group (Figure 4).

**Figure 5:**
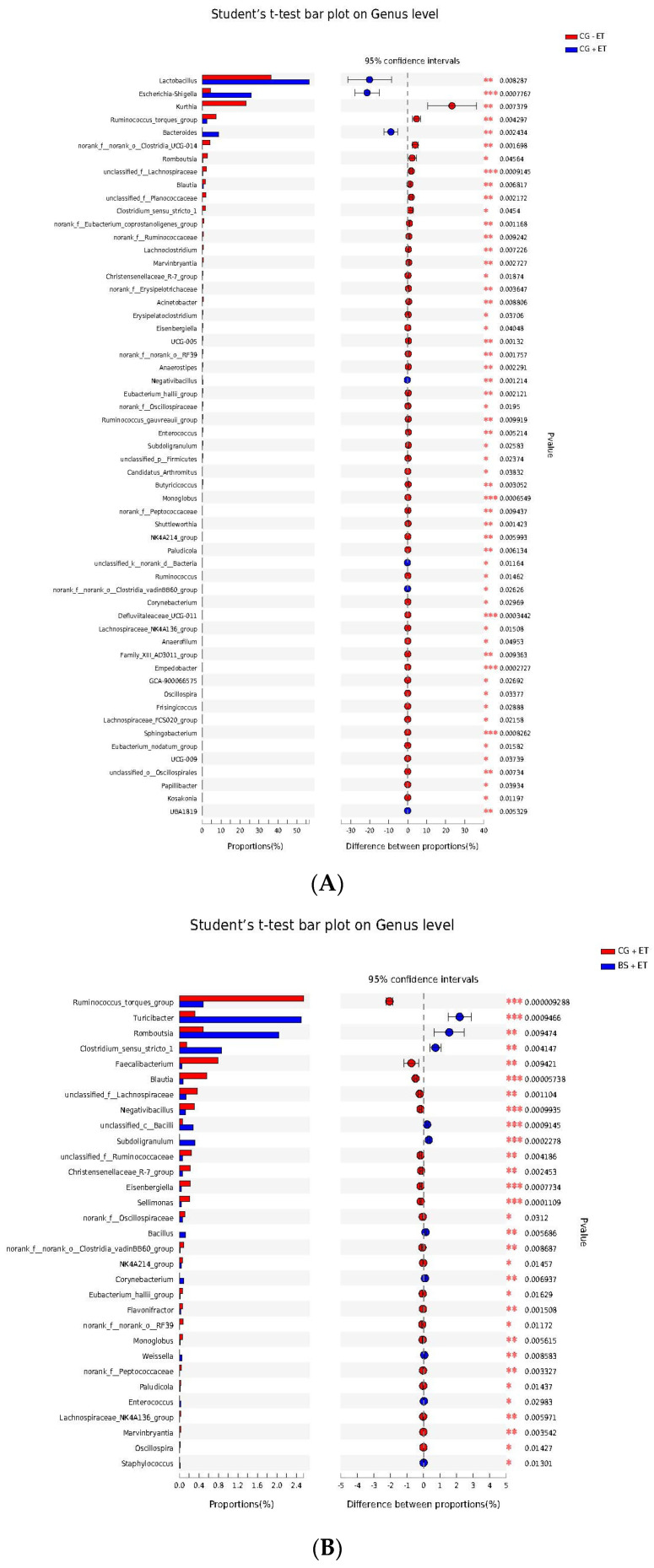

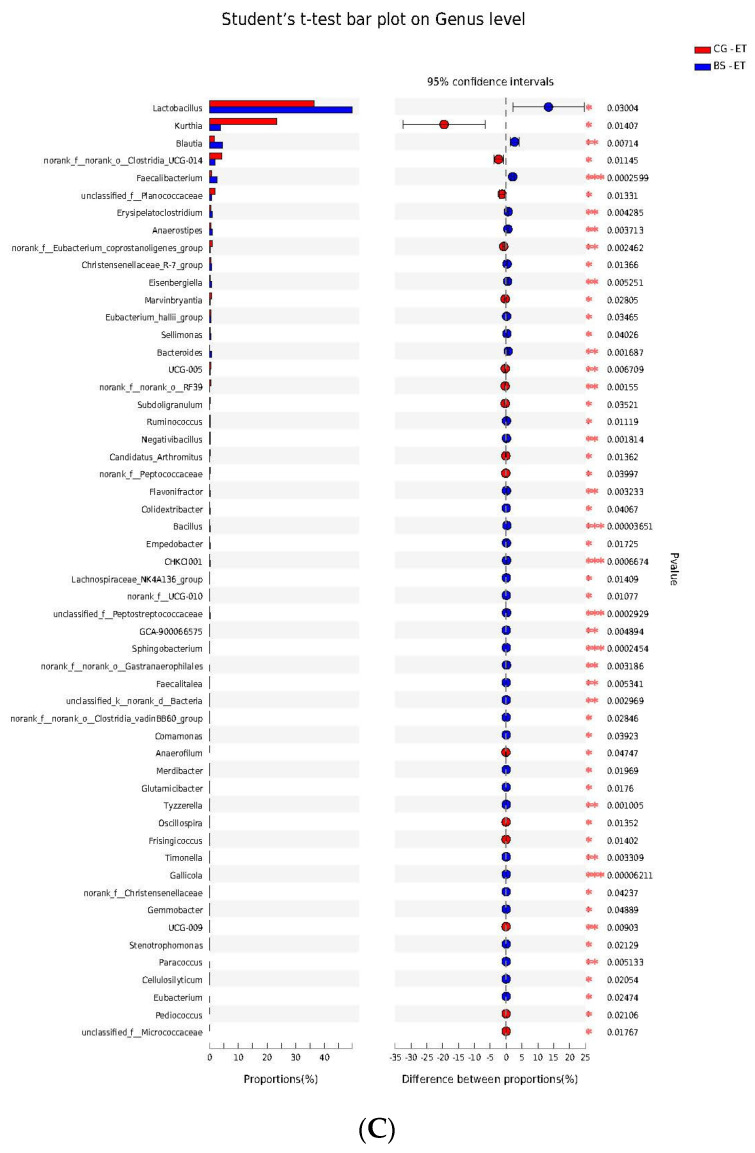
Microbial abundances enriched in treatments at genus level. (**A**) Enriched abundances of taxa at genus in groups CG − ET versus CG + ET comparison. (**B**) Enriched abundances of taxa at genus in groups CG + ET versus BS + ET comparison. (**C**) Enriched abundances of taxa at genus in groups CG − ET versus BS − ET comparison. *, **, *** represent significant difference.

An LEfSe analysis was also performed to fully understand the influence of probiotic on gut microbiota during *Eimeria* infection. The results of LEfSe analysis showed that several genera were affected by the addition of probiotic and inoculation of *Eimeria* infection (Figure 5, Table S2). Compared to CG − ET, the non-treated infected control group significantly declined in their abundances of *Kurthia*, *Ruminococcus torques group*, *Blautia*, *Lachnoclostridium*, *Marvinbryantia*, *Christensenellaceae R-7 group*, *Acinetobacter*, *Eisenbergiella*, *UCG-005*, *Anaerostipes*, *Eubacterium hallii group*, *Ruminococcus gauvreauii group*, *Candidatus Arthromitus*, *Butyricicoccus*, *Monoglobus*, *Shuttleworthia*, *NK4A214 group*, *Paludicola*, *Ruminococcus*, *Defluviitaleaceae UCG-011*, *Lachnospiraceae NK4A136 group*, *Anaerofilum*, *Family XIII AD3011 group*, *Empedobacter*, *GCA-900066575*, *Oscillospira*, *Frisingicoccus*, *Lachnospiraceae FCS020 group*, *Sphingobacterium*, *Eubacterium nodatum group*, *UCG-009*, *Papillibacter*, *Kosakonia*, *UBA1819*, *Romboutsia*, *Subdoligranulum*, *Enterococcus*, *Clostridium sensu stricto 1*, *Corynebacterium* and some unclassified and norank genera, while increased abundances of *Lactobacillus*, *Escherichia-Shigella*, *Bacteroides Negativibacillus* and *UBA1819* were detected (Figure 5A, Table S2). Probiotic-treated and challenged chickens on the other hand restored (increased) the abundances of *Clostridium sensu stricto 1*, *Corynebacterium*, *Enterococcus*, *Romboutsia* and *Subdoligranulum* and decreased the abundances of *Faecalibacterium*, *Lachnoclostridium*, *Eisenbergiella*, *Sellimonas*, *Flavonifractor*, *Monoglobus*, *Lachnospiraceae*, *NK4A136-group*, *NK4A214-group*, *Blautia*, *Ruminococcus torques-group*, *Christensenellaceae R-7-group*, *Eubacterium hallii-group* and *Paludicola* compared to the *Eimeria*-infected non-treated group (Figure 5B, Table S2). More importantly, the abundances of some genera associated with the *Bacilli* class were found to be enriched in challenged chickens fed a probiotic diet compared to challenged chickens fed a normal diet. They included *Bacillus*, *Weissella*, *Staphylococcus*, *Bacilli* unclassified and *Turicibacter* (Figure 5B, Table S2).

In this experiment, we also performed the LEfSe analysis in the comparison of BS + ET versus CG + ET and the results are presented in Figure 5C and Table S2.

### 3.5. Predicted Functions of Microbiota

**Figure 6:**
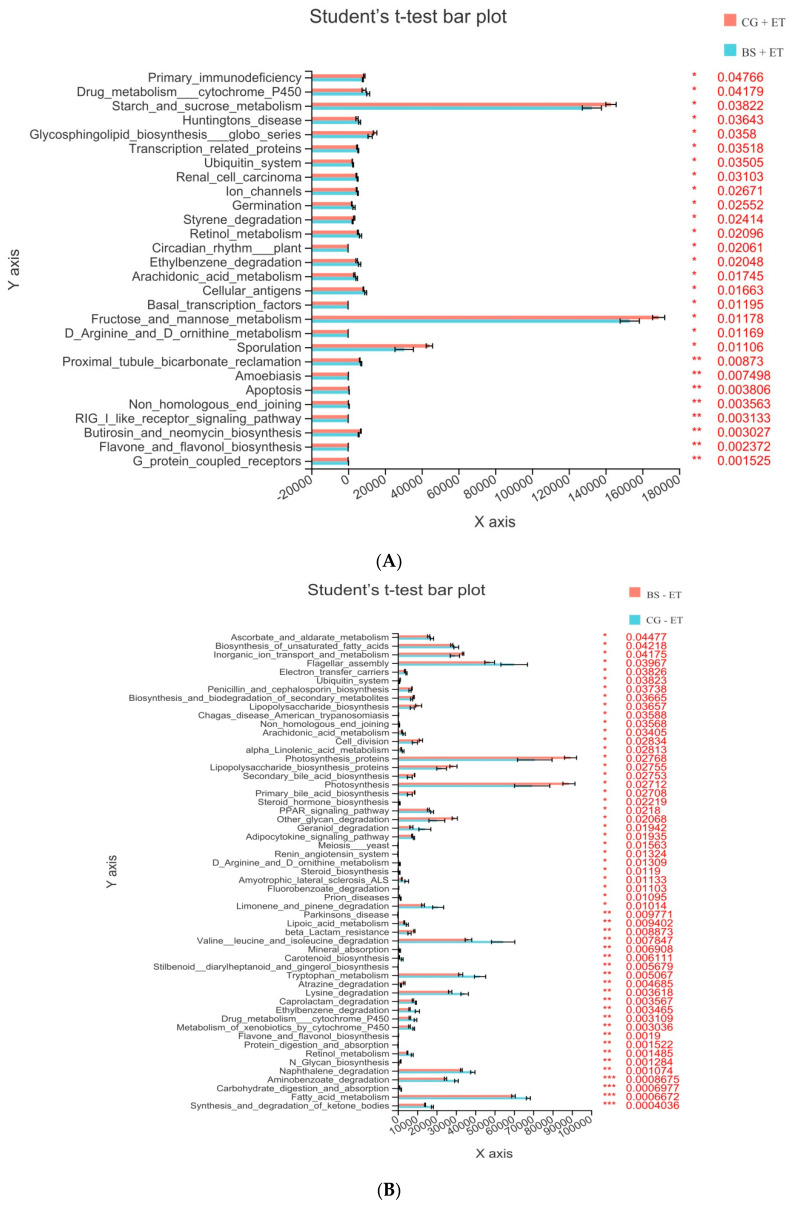
Third-level KEGG pathways affected by probiotic feeding and *Eimeria* infection. (**A**) Affected KEGG pathways in groups CG + ET versus BS + ET comparison. (**B**) Affected KEGG pathways in groups CG − ET versus BS − ET comparison. *, **, *** represent significant difference.

To predict the microbial community functions affected by the addition of probiotic and inoculation of *Eimeria* infection, a PICRUSt analysis was carried out using KEGG databases. Third-level KEGG analysis revealed that 17 KEGG pathways were significantly affected (up-regulated) by the challenged chickens fed a probiotic diet when compared with challenged chickens fed a normal diet (Figure 6A, Table S3). On the other hand, 23 KEGG pathways were significantly enriched in non-challenged chickens fed probiotic diet in comparison of non-challenged chickens fed normal diet (Figure 6B, Table S3).

## 4. Discussion

Coccidiosis is one of the most significant ubiquitous poultry diseases globally that causes severe destruction of the intestinal tract, resulting in reduced growth performance and immunity, disrupted gut homeostasis and perturbed gut microbiota [3,29]. The supplementation of probiotics (as an alternative of antibiotics and anti-coccidials) has been observed to play a key role in improving growth performance, enhancing innate and adaptive immunity, maintaining gut homeostasis and modulating microbiota during enteric infection [30]. In our previous study, we examined the prophylactic efficacy of *B. subtilis* probiotic on body weight, oocyst shedding in feces, bloody diarrhea scores, cecal lesion scores and whole transcriptome responses of chicken under *E. tenella* infection [23]. This study was then designed to investigate the chicken gut whole microbiota responses to *B. subtilis* probiotic feeding under induced *Eimeria* infection. Our results for alpha diversity showed that the CG + ET group showed decreased Ace, Chao 1 and Shannon, but decreased Simpson indexes compared to CG − ET. These findings imply that the enteric infections negatively influence species’ richness (Ace and Chao 1) as well as bacterial diversity (Shannon). Probiotic-fed challenged chickens on the other hand also showed no significant effects on bacterial richness and biodiversity compared to control positive untreated challenged chickens. Similar findings were reported by Guo et al. [31], who found that chickens fed *Bacillus*-based probiotic experienced no significant effects on bacterial richness and biodiversity compared to chickens in the positive control group during enteric infection.

Abundances of the taxa *Firmicutes*, *Proteobacteria* and *Bacteroidota* at the phylum level, while *Lactobacillus* at the genus level were found to be dominant in chickens [32,33]. Similar findings were also noticed in the present experiment. Meanwhile, challenged birds in groups CG + ET and BS + ET had lower abundance of *Firmicutes* and higher abundances of *Proteobacteria* and *Bacteroidota* than that of birds in the control non-treated non-challenged group and *B. subtilis*-fed non-challenged group. Contrary to our results, Chen et al. [33] reported that *Eimeria* infection significantly decreased the abundance of *Firmicutes* and increased the abundances of *Proteobacteria* and *Bacteroidota* in broilers when compared with unchallenged broilers. *Firmicutes* and *Bacteroidota* play a key role in nutrient absorption and gut homeostasis in chickens [34]. A higher proportion of *Firmicutes* could suppress pathogenic microbes, restore homeostasis and increase nutrient absorption, whereas an increase in *Bacteroidota* phylum could lead lower nutrient absorption and dysbiosis [35]. The severity of enteric infection leads to an increase the abundances of phyla *Proteobacteria* and *Bacteroidota* and a decreased abundance of phyla *Firmicutes* [36]. Compared to the negative control group, the decreased abundance of *Firmicutes* and increased abundances of *Proteobacteria* and *Bacteroidota* due to *Eimeria* infection herein imply the negative effects of coccidia on microbiota balance.

*Lactobacillus* is an important commensal genus in the chicken gastrointestinal tract [37], which provides beneficial effects on the health and performance by producing antimicrobial substances [37], short chain fatty acids, exopolysaccharides and additional sources of energy [38]. In the present study, challenged untreated chickens and probiotic-fed chickens in the presence and absence of coccidia infection tended to increase the abundance of the *Lactobacillus* genus compared to control chickens untreated unchallenged. However, the BS + ET group had no significant influence on the taxa *Firmicutes*, *Proteobacteria*, *Bacteroidota* (at phylum level) and *Lactobacillus* (at genus level) compared to CG + ET, while BS − ET on the taxa *Firmicutes*, *Proteobacteria* and *Bacteroidota* was compared to CG − ET.

To understand the deep mechanism of *Eimeria* infection and probiotic feeding on bacterial genera, we performed an LEfSe analysis. Our results revealed that *Eimeria* challenged untreated chickens decreased the abundances of *Kurthia*, *Ruminococcus torques group*, *Blautia*, *Lachnoclostridium*, *Marvinbryantia*, *Christensenellaceae R-7 group*, *Acinetobacter*, *Eisenbergiella*, *UCG-005*, *Anaerostipes*, *Eubacterium hallii group*, *Ruminococcus gauvreauii group*, *Candidatus Arthromitus*, *Butyricicoccus*, *Monoglobus*, *Shuttleworthia*, *NK4A214_group*, *Paludicola*, *Ruminococcus*, *Defluviitaleaceae UCG-011*, *Lachnospiraceae NK4A136 group*, *Anaerofilum*, *Family XIII AD3011 group*, *Empedobacter*, *GCA-900066575*, *Oscillospira*, *Frisingicoccus*, *Lachnospiraceae FCS020 group*, *Sphingobacterium*, *Eubacterium nodatum group*, *UCG-009*, *Papillibacter*, *Kosakonia*, *UBA1819*, *Romboutsia*, *Subdoligranulum*, *Enterococcus*, *Clostridium sensu stricto 1*, *Corynebacterium* and some unclassified and norank genera compared to control chickens. On the other hand, probiotic fed chickens increased the abundances of some *Eimeria*-affected genera (*Romboutsia*, *Subdoligranulum*, *Enterococcus*, *Clostridium sensu stricto 1*, *Corynebacterium*) along with some commensal genera belonging to the *Bacilli* class, including *Bacillus*, *Weissella*, *Staphylococcus*, *Bacilli* unclassified and *Turicibacter*. It is well known that host gut microbiota plays significant roles in regulating immunity and maintaining several metabolic processes by triggering metabolites [39]. During enteric infection, gut microbiota induce protective responses against pathogens, maintain immunoregulatory pathways against antigens and suppress inflammatory responses [40]. The genus *Romboutsia* is the group of commensal bacteria [41], which function in metabolic reactions of the host in terms of carbohydrate utilization, single amino acids fermentation, anaerobic respiration and end products of metabolic process [42]. The genera *Subdoligranulum* and *Clostridium sensu stricto 1* were found to be involved in butyrate production [43,44]. The production of butyrate by these commensal genera then confers protective mechanisms by ameliorating mucosal inflammatory reactions and oxidative status, supporting the integrity of epithelial barrier and modulating intestinal motility [45]. Species of the *Enterococcus* are used as probiotics and have been reported to produce antimicrobial peptides, such as bacteriocins [46]. Members of the class *Bacilli*, including *Bacillus*, *Weissella*, *Staphylococcus*, *Bacilli* unclassified and *Turicibacter* contribute to metabolism of food and drugs particles, help in maintaining host health status and gut homeostasis [47]. Taking these reports as evidence, it can be assumed from our results that the feeding of probiotic possibly alleviated the negative effects of *Eimeria* by stimulating the activities of these commensal genera, which may in turn lead to the stimulation of immune pathways, reduction of inflammatory responses and oxidative status, maintenance of gut barrier integrity and *Eimeria* perturbation of metabolic processes.

## 5. Conclusions

This study was focused on the the role of *B. subtilis* probiotic on gut microbiota in the presence and absence of *Eimeria* infection. Our results demonstrated that the *B. subtilis*-based probiotic can increase the abundance of commensal bacterial populations. This may in turn lead to an increase in butyrate production, modulate the anti-inflammatory function, anti-oxidative and metabolites factors and trigger the protective pathways against pathogens. Moreover, a fecal microbiome analysis explored the taxonomic and functional signatures of affected microbial profiles in response to probiotic feeding in the presence and absence of coccidia infection. Future studies should be carried out to explore the individual and combined mechanisms of different *Bacillus*-based probiotic strains on whole Transcriptome and microbiome analysis.

## Figures and Tables

**Table 1:** Quality control parameters.

Sample	Sequence Number	Base Number	Mean Length	Good’s Coverage
CG − ET_1	50120	21,043,414	419.86	0.99
CG − ET_2	47657	20,114,212	422.06	0.99
CG − ET_3	49749	20,880,643	419.71	0.99
CG + ET_1	39661	16,889,906	425.85	0.99
CG + ET_2	38967	16,588,260	425.70	0.99
CG + ET_3	41425	17,642,574	425.89	0.99
BS − ET_1	42819	17,960,932	419.46	0.99
BS − ET_2	47562	19,942,902	419.30	0.99
BS − ET_3	47540	19,961,785	419.89	0.99
BS + ET_1	41516	17,708,570	426.54	0.99
BS + ET_2	42939	18,320,028	426.65	0.99
BS + ET_3	41109	17,562,370	427.21	0.99

## Data Availability

The raw data associated with this study were deposited into the NGDC database (accession: CRA004666).

## Supplementary Materials

The following supporting information can be downloaded at: https://www.mdpi.com/article/10.3390/microorganisms10081548/s1, Table S1: Alpha diversity index; Table S2: Affected taxa at genus level by the treatments; Table S3: Affected KEGG pathways by the treatments.
